# Evaluation of imaging findings and prognostic factors after whole-brain radiotherapy for carcinomatous meningitis from breast cancer: A retrospective analysis

**DOI:** 10.1097/MD.0000000000021333

**Published:** 2020-07-31

**Authors:** Yukinori Okada, Tatsuyuki Abe, Mio Shinozaki, Akiko Tanaka, Mariko Kobayashi, Gomi Hiromichi, Yoshihide Kanemaki, Naoki Nakamura, Yasuyuki Kojima

**Affiliations:** a Department of Radiology; b Department of Surgery, Division of Breast and Endocrine Surgery, St. Marianna University School of Medicine, Kawasaki, Kanagawa, Japan.

**Keywords:** carcinomatous meningitis from breast cancer, subtype, whole-brain radiotherapy

## Abstract

This study aimed to evaluate the imaging findings and prognostic factors after whole-brain radiotherapy in patients with carcinomatous meningitis from breast cancer.

A retrospective analysis of imaging data and prognostic factors was performed in patients treated with whole-brain radiotherapy or whole-brain/spine radiotherapy immediately after the first diagnosis of carcinomatous meningitis from breast cancer at our hospital from January 1, 2010 to December 31, 2018. Statistical significance was set at *P* < .05 (two-tailed).All patients (n = 31) were females with the mean age of 58.0 ± 11.0 years. The breast cancer subtypes were luminal (n = 14, 45.1%), human epidermal growth factor receptor 2 (HER2)-positive (n = 9, 29.0%), and triple-negative (n = 8, 26.0%) breast cancer. Brain metastasis and abnormal contrast enhancement in the sulci were observed in 21 (67.7%) and 24 (80.6%) patients, respectively. The median survival time after cancerous meningitis diagnosis was 62 (range, 6–657) days. Log-rank test showed significant differences in median survival time after cancerous meningitis diagnosis: 18.0 days for subjects treated with 30 Gy in < 10 fractions (n = 7) vs 78.5 days for subjects treated with 30 Gy in ≥10 fractions (n = 24) (*P* < .01) and 23.0 days for the triple-negative subtype vs 78.5 days for the other subtype (*P* < .01) groups. Univariate analysis using the Cox regression model showed significant differences in median survival time after cancerous meningitis diagnosis between the group treated with 30 Gy in <10 fractions and the group treated in ≥10 fractions (hazard ratio [HR] 0.08, 95% confidence interval [CI], 0.03–0.26; *P* < .01), and between the triple-negative subtype and the other subtypes (HR = 5.48; 95% CI, 1.88–16.0; *P* < .01) groups.

Discontinuation of whole-brain radiotherapy and the presence of triple-negative breast cancer were indicators of poor prognosis.

## 1 Introduction

In Japan, breast cancer is the most common malignancy affecting females^[1]^ and is associated with frequent metastasis to the brain, lungs, liver, and bones.^[2]^ Subtypes of breast cancer are classified by the presence or absence of hormone receptors, human epidermal growth factor receptor 2 (HER2), or the proliferative activity of cancer cells. The commonly researched subtypes include luminal-A, luminal-B, HER2-positive, and triple-negative breast cancers. The potential for distant metastasis to various organs differs by the subtype of breast cancer, for example, luminal types metastasize to bones whereas HER2-positive and triple-negative breast cancers metastasize to solid organs (brain, lung, and liver).

Whole-brain radiotherapy is performed for brain metastasis from breast cancer. However, cognitive decline occurs around 2 years after whole-brain radiation.^[3]^ Since the survival time is similar between whole-brain radiotherapy and stereotactic radiotherapy for patients with ≤10 metastatic brain lesions, in such scenarios stereotactic radiotherapy is often used as a measure to avoid cognitive decline.^[4]^ Carcinomatous meningitis is the pathogenic mechanism by which breast cancer cells extend to the brain surface, subarachnoid space, ventricles, and cisterns via cerebrospinal fluid. Some patients with carcinomatous meningitis exhibit central nervous system signs. Imaging studies—computed tomography (CT), magnetic resonance imaging (MRI)—report heterogeneous contrast enhancement in the base of the brain in some patients with carcinomatous meningitis and attribute it to gravity. While the reported incidence of carcinomatous meningitis in breast cancer patients is about 3.5%,^[5]^ only a few studies provide accurate incidences based on imaging findings. In addition, the prognostic factors of carcinomatous meningitis have not been thoroughly explored. This study aimed to evaluate the imaging findings and prognostic factors of carcinomatous meningitis from breast cancer.

## 2 Methods

### 2.1 Case selection

This single-center study retrospectively evaluated the electronic medical records and radiation records of patients treated with whole-brain radiotherapy or whole-brain/spine radiotherapy immediately after the first diagnosis of carcinomatous meningitis from breast cancer in our hospital from January 1, 2010 to December 31, 2018.

### 2.2 Imaging findings

Data collected from brain CT (16 or 64 multi-slice CT, Cannon Medical, Otawara, Japan) or MRI (Inginia 3.0T, Achieva nova Dual 1.5T, Achieva 1.5T; Philips, Tokyo, Japan or Galan 3T; Cannon Medical, Otawara, Japan) included

1. the presence or absence and the number of brain metastases and brain metastases number, size;
2. the presence or absence of abnormal contrast sensitivity along the cerebral and cerebellar sulci;
3. the presence or absence of abnormal contrast sensitivity in the dura mater; and
4. the presence or absence of abnormal contrast sensitivity in the head organs.

### 2.3 Laboratory investigations and follow-up

Data on white blood cell count, red blood cell count, hemoglobin count, platelet count, total protein, albumin, C-reactive protein (CRP), carcinoembryonic antigen (CEA), and carbohydrate antigen 15–3 (CA15-3) performed before whole-brain radiotherapy were collected. Histopathologic data included the histologic types, pathological findings, and subtypes of the primary breast cancer tumors. The presence or absence of radiotherapy discontinuation was evaluated in patients.

### 2.4 Prognostic factors

The following factors were analyzed for their prognostic value after whole-brain irradiation:

1. age,
2. histological type,
3. pathological findings,
4. subtype,
5. presence or absence of other metastases,
6. blood test results (white blood cell count, red blood cell count, hemoglobin count, platelet count, total protein, albumin, and CRP),
7. dose of whole-brain radiotherapy,
8. presence or absence of discontinued radiotherapy, and
9. CT or MRI findings.

Day 1 was defined as the day of diagnosis of cancerous meningitis based on cerebrospinal fluid cytology, CT, or MRI findings. The day of discontinuation was defined as the day of death. The last observation day was December 31, 2018.

### 2.5 Statistical analysis

All statistical analyses were performed using the statistical software EZR developed by the Jichi Medical University Saitama Medical Center (Jichi Medical University Omiya Medical center). Kaplan–Meier analysis (log-rank test), and Cox proportional-hazard model were used for the evaluation of prognostic factors. *P* values < .05 were considered statistically significant.

### 2.6 Ethical considerations

This study was approved by the ethics committee of the St. Marianna University School of Medicine (approval number: 4337). For ethical considerations, patients were informed of the purpose of the study with the option to opt-out through the hospital homepage and in the hospital.

## 3 Results

From January 1, 2010 to December 31, 2018, 31 patients received their first diagnosis of carcinomatous meningitis and underwent immediate whole-brain radiotherapy. All patients were female with a mean age of 58.0 ± 11.0 years (Table 1).

**Table 1:**
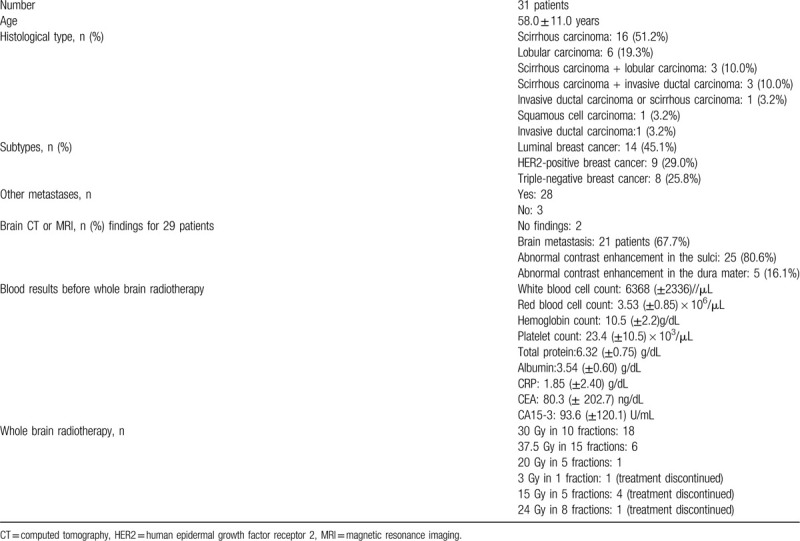
Patients results.

### 3.1 Histological type and subtype

Histological diagnoses included scirrhous carcinoma (n = 16, 51.2%), lobular carcinoma (n = 6, 19.3%), scirrhous and lobular carcinoma (n = 3, 10.0%), scirrhous and invasive ductal carcinoma (n = 3, 10.0%), invasive ductal or scirrhous carcinoma (n = 1, 3.2%), squamous cell carcinoma (n = 1, 3.2%), and invasive ductal carcinoma (n = 1, 3.2%). All carcinoma containing or contained in scirrhous carcinoma were classified as scirrhous carcinoma for analysis purposes (Table 1).

Subtype diagnosis included luminal breast cancer (n = 14, 45.1%), HER2-positive breast cancer (n = 9, 29.0%), and triple-negative breast cancer (n = 8, 25.8%). Other metastasis occurred in 28 (90.3%) patients. Three patients required special consideration of classification: one patient with bilateral breast cancer (right T2N1M0 triple-negative breast cancer and left T1N0M0 luminal breast cancer) was classified as having triple-negative breast cancer according to the staging system. Another patient with T1N2M0 triple-negative primary breast cancer was classified as having HER2-positive breast cancer because lymph node and breast tumor recurrence at 1 year were HER2 positive based on fluorescence in situ hybridization (FISH) findings. Yet another patient with T1N0M0 triple-negative primary breast cancer was classified as having luminal breast cancer because the patient was diagnosed with luminal breast cancer on re-examination and received hormone therapy after surgery for the primary tumor (Table 1).

### 3.2 Other metastases

In 28 patients (90.3%), metastasis to other sites was present at the onset of cancerous meningitis. Twenty-one (67.7%) patients (5 with liver metastasis, 5 with lymph node metastasis) had bony metastasis. Four (12.9%) patients had lymph node metastasis, while one (3.2%) patient had lung and lymph node metastases. one (3.2%) patient had lung and liver metastases. Three (9.7%) patients had no other metastases (Table 1).

### 3.3 Imaging findings

All 31 patients underwent either a brain CT or MRI, and the findings were not evaluable in two patients. Of the remaining 29 patients, 26 underwent brain MRI, and 3 patients underwent brain CT. Brain metastasis were observed in 21 patients (single: 6, multiple: 15) (67.7%) and only one patient show the maximum brain metastasis diameter was above 3 cm. Twenty patient show the maximum brain metastasis diameter was under 3 cm. Abnormal contrast enhancement in the sulci were observed in 25 (80.6%) patients. Abnormal contrast enhancement in the dura mater was observed in 5 (16.1%) patients (Table 1).

### 3.4 Results of the blood test

All measurements were performed immediately before whole-brain radiotherapy. The mean values of white blood cell count, red blood cell, count, hemoglobin count, and platelet count measured in 29 patients were 6368 (±2336)/μL, 3.53 (±0.85) × 106/μL, 10.5 (±2.2) g/dL, and 23.4 (±10.5) × 103/μL, respectively. The mean total protein level in 27 patients was 6.32 (±0.75) g/dL. The mean albumin level measured in 20 patients was 3.54 (±0.60) g/dL and 7 patients show above 3.9 g/dL (cut off value). The mean CRP level measured in 21 patients was 1.85 (±2.40) g/dL. The mean CEA and CA15-3 levels measured in 24 patients were 80.3 (±202.7) ng/dL and 93.6 (±120.1) U/mL, respectively (Table 1).

### 3.5 Whole-brain radiotherapy

Complete whole-brain radiotherapy dosage received was 30 Gy in 10 fractions for 18 patients, 37.5 Gy in 15 fractions for 6 patients, and 20 Gy in 5 fractions for 1 patient. The remaining 6 patients discontinued whole-brain radiotherapy at a dose of 3 Gy in 1 fraction (n = 1) or 15 Gy in 5 fractions (n = 4) or 24 Gy in 8 fractions (n = 1) (Table 1).

Elekta Synergy (Cannon Medical System; Ohtawara) and Primus (Cannon Medical System; Ohtawara) were used for extra beam irradiation therapy. And Mevatron (Cannon Medical System; Ohtawara) was used for extra beam irradiation therapy until 2012.

### 3.6 Prognostic factors

#### 3.6.1 All patients

**Figure 1:**
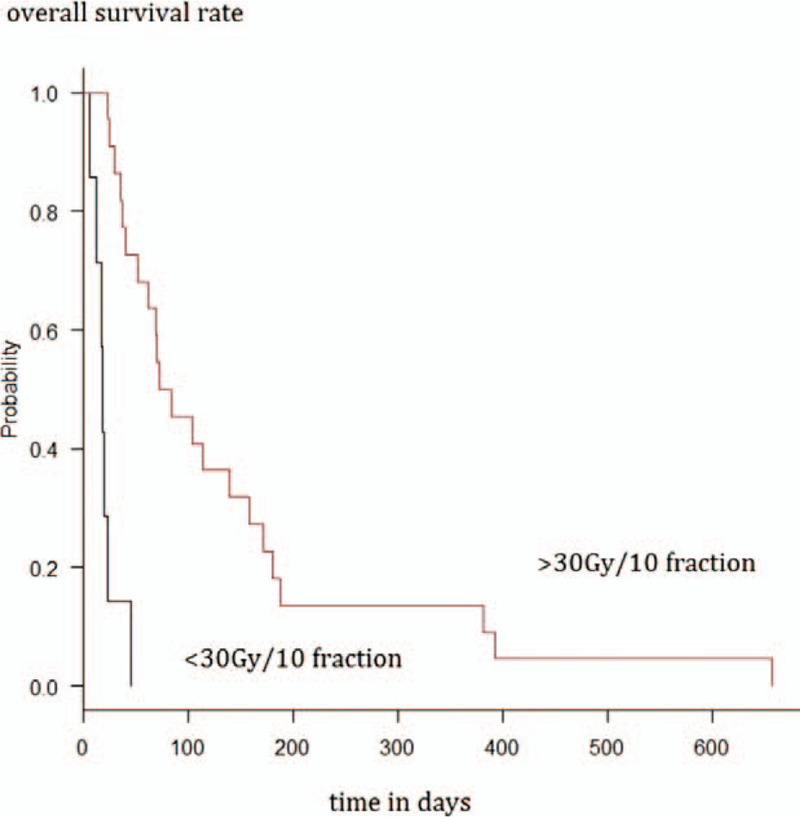
Survival analysis of patients treated with 30 Gy in <10 fractions and with 30 Gy in ≥10 fractions using the Kaplan–Meier method.

Out of the total of 31 patients, 29 were followed up, and all died during the follow-up period. Median survival time after post-cancerous meningitis diagnosis was 62 (range, 6–657) days. Log-rank test showed a significant difference (*P* < .01) in the post-diagnosis median survival time between the group treated with 30 Gy in <10 fractions (n = 7, 18.0 days, 95% confidence interval [CI] = 6–23 days) and the group treated with 30 Gy in ≥10 fractions (n = 22, 78.5 days, 95% CI = 37–158 days) (Fig. 1).

**Figure 2:**
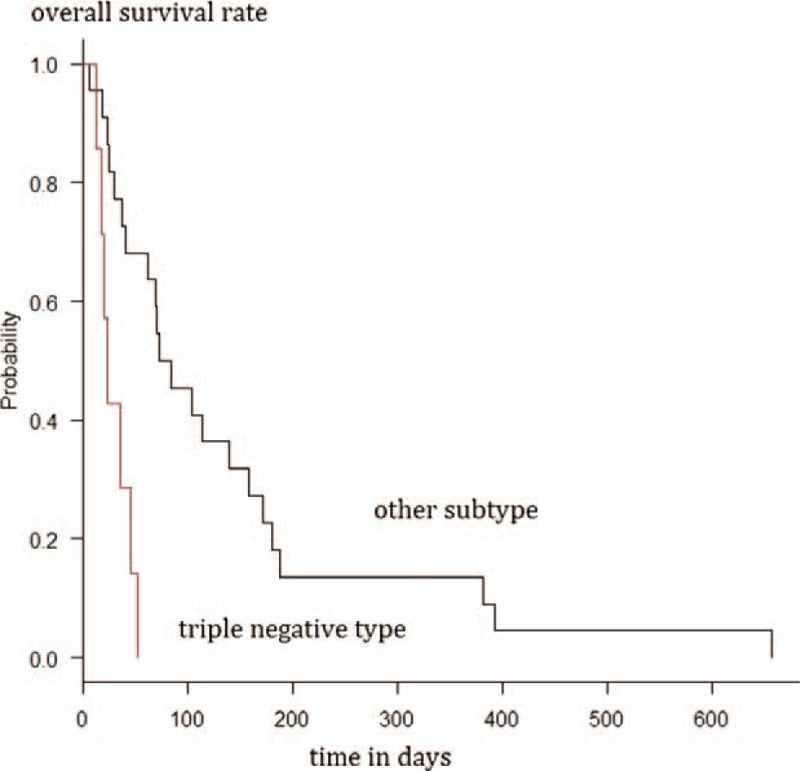
Survival analysis of patients with triple-negative and other subtypes of breast cancer using the Kaplan–Meier method.

After excluding the three patients requiring special consideration of classification, the log-rank test showed a significant difference (*P* < .01) in the post-diagnosis median survival time between the triple-negative breast cancer group (n = 6, 29.5 days, 95% CI = 17–not available days) and the other subtype group (n = 20, 78.5 days, 95% CI = 29–158 days). The difference in post-diagnosis mean survival time remained significant (*P* < .01) even with the inclusion of the three patients requiring special consideration of classification: triple-negative breast cancer group (n = 7, 23.0 days, 95% CI = 12–46 days) vs the other subtype group (n = 22, 78.5 days, 95% CI = 37–158 days) (Fig. 2).

**Figure 3:**
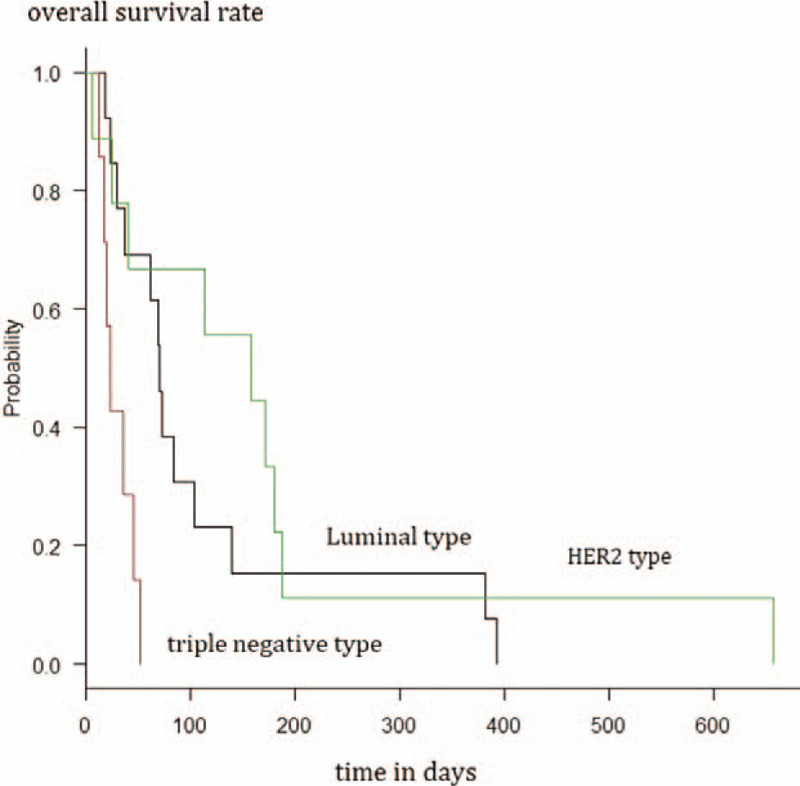
Survival analysis of patients with luminal type, triple-negative and HER2 type of breast cancer using the Kaplan–Meier method. HER2 = human epidermal growth factor receptor 2.

The log-rank test that included the three patients requiring special consideration of classification, showed a significant difference (*P* < .01) in the post-diagnosis median survival time among the luminal breast cancer group (n = 13, 70.0 days, 95% CI = 29–104 days), triple-negative breast cancer group (n = 7, 23.0 days, 95% CI = 12–46 days), and HER2-positive group (n = 9, 158 days, 95% CI = 6–187 days) (Fig. 3).

Univariate analysis using the Cox regression model showed a significant difference in the median survival time between the group treated with 30 Gy in <10 fractions and the group treated with 30 Gy in ≥10 fractions (hazard ratio [HR] = 0.08, 95% CI = 0.03–0.26; *P* < .01). Univariate analysis using the Cox regression model showed a significant difference in the median survival time between the triple-negative breast cancer group and the other subtype group without exclusion (HR = 5.48; 95% CI, 1.88–16.0; *P* < .01) and with exclusion (HR = 4.41, 95% CI = 1.46–13.4; *P* < .01) of the three patients requiring special consideration of classification. There were no significant differences between the groups by other factors (Table 2).

**Table 2:**
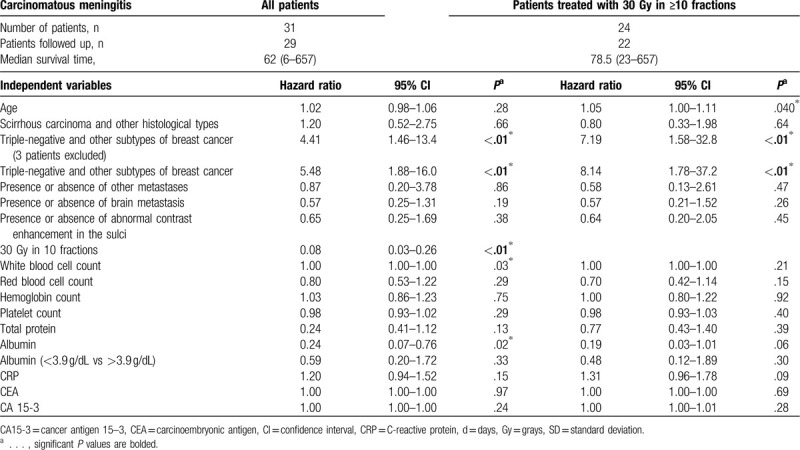
Prognostic factors of overall survival based on diagnosis of carcinomatous meningitis.

#### 3.6.2 Patients treated with 30 Gy in ≥10 fractions

Of 24 patients treated with 30 Gy in ≥10 fractions, 22 were followed up, and all died during the follow-up period. The median survival time after the diagnosis of cancerous meningitis was 78.5 (range, 23–657) days. The log-rank test after excluding the three patients requiring special consideration of classification showed a significant difference in the post-diagnosis median survival time between the triple-negative breast cancer group (n = 3, 36 days, 95% CI = 23–not available days) and the other subtype group (n = 17, 104 days, 95% CI = 40–180 days) (*P* < .01). Even with the inclusion of the three patients requiring special consideration of classification, the significant difference in the post-diagnosis median survival time between the triple-negative breast cancer group (n = 3, 36 days, 95% CI = 23–not available days) and the other subtype group (n = 19, 104 days, 95% CI = 62–172 days) remained (*P* < .01).

The log-rank test that included the one patient requiring special consideration of classification showed a significant difference (*P* < .01) in the post-diagnosis median survival time among the luminal breast cancer group (n = 11, 73.0 days, 95% CI, 37–139 days), triple-negative breast cancer group (n = 3, 36.0 days, 95% CI = 23–not available days), and HER2-positive group (n = 8, 165 days, 95% CI = 25–187 days).

Univariate analysis using the Cox regression model showed a significant difference in the median survival time about age (HR = 1.05, 95%CI = 1.00–1.11, *P* < .01). Univariate analysis using the Cox regression model showed a significant difference in the median survival time between the triple-negative breast cancer group and the other subtype group without exclusion (HR = 8.14, 95% CI = 1.78–37.2, *P* < .01), and with exclusion (HR = 7.20, 95% CI = 1.58–32.9, *P* < .01) of the three patients requiring special consideration of classification (Table 2), There were no significant differences between the groups in other factors.

## 4 Discussion

This retrospective single-center study shows a high incidence (n = 25, 80.1%) of abnormal sulcal contrast enhancement in the CT or MRI of patients with carcinomatous meningitis, most commonly in the cerebellar hemispheres. The slow flow of cerebrospinal fluid due to gravity in the cerebellar hemispheres may explain this imaging finding in breast cancer carcinomatosis. Thus, the examination of contrast enhancement in the cerebellar sulci may improve diagnostic performance in carcinomatous meningitis. In this study of 31 patients with carcinomatous meningitis, 28 patients (90.3%) had other metastasis suggesting that carcinomatous meningitis occurs during the terminal phase of breast cancer.

Even with various treatment modalities such as intrathecal injection, whole-brain radiotherapy, and whole-brain/spine radiotherapy, the median survival time after carcinomatous meningitis diagnosis is about 77 days.^[5]^ Overall, the median survival time is 4 to 6 weeks without treatment and 3 to 6 months with treatment.^[6]^ In this study, the median survival time after cancerous meningitis diagnosis was 62 (range, 6–657) days with whole-brain radiotherapy, and the duration increased with an increased number of radiation fractions. The survival time in our patients was rather weak compared with that reported in previous studies. Although the reason is not entirely apparent, one explanation is the use of more effective treatment methods in previous studies.

Currently, no established treatment guidelines exist for carcinomatous meningitis from breast cancer. The National Comprehensive Cancer Network (NCCN) Guidelines for Breast Cancer recommends determining the treatment policy based on the criteria for good-risk (Karnofsky performance status [KPS] ≥60, no severe complications, few metastases, effective systematic treatment) and poor-risk disease (KPS < 60, multiple severe neurological complications, giant central nervous system lesions, cerebral disorders, encephalopathy, extensive systematic metastasis, few available treatment options).^[7]^ The classification is somewhat subjective; moreover, the guidelines recommend using whole-brain or whole-brain/spine radiotherapy in both good- and poor-risk disease,^[7]^ and a recommended dose of 30 Gy in 10 fractions to 36 Gy in 12 fractions.^[8]^ However, in contrast to the NCCN Guidelines for Breast Cancer, the presence or degree of metastatic lesions in the brain or other systems did not affect the prognosis in this study. The histological type of breast carcinoma affect the prognosis.^[9]^ High incidence of brain metastasis is reported for triple-negative breast cancer and HER2 breast cancer subtypes.^[2]^ Although the histological type did not affect prognosis in this study.^[10]^ Furthermore, in this study of 30 patients with carcinomatous meningitis, the ratio of luminal breast cancer to HER2 or triple-negative breast cancer was about 1:1 (i.e., 14 patients had luminal breast cancer, 8 had HER2 breast cancer, and 9 had triple-negative breast cancer), suggesting that breast cancer subtypes may not affect the incidence of carcinomatous meningitis.

The median survival time of patients with multiple brain metastasis has been reported to be 23.1 months for luminal-A, 15.0 months for luminal-B, 12.5 months for HER2, and 6.4 months for triple-negative breast cancer.^[10]^ Poor prognosis is reported for brain metastasis treated with stereotactic radiotherapy in patients with triple-negative or HER2 breast cancer.^[11]^ Other studies report a good prognosis for HER2 breast cancer with brain metastasis^[12]^ and poor prognosis in triple-negative breast cancer with brain metastasis.^[13,14]^ A large-scale study of brain metastasis identified tumor size, multiple metastases, non-brain parenchymal lesions, triple-negative or HER2-positive breast cancer, and poor KPS as poor prognostic factors.^[15]^ In this study, all patients with triple-negative breast cancer showed markedly poor prognosis than other subtypes, even with radiotherapy completion (30 Gy in <10 fractions). In line with other studies, we attribute this finding to the low radiosensitivity of triple-negative breast cancer cells. Another reason could be the more frequent distant metastasis seen in patients with triple-negative breast cancer.

Local recurrence occurs more commonly with HER2 breast cancer, while new brain metastasis is more common with triple-negative breast cancer.^[16]^ In this study, most patients had other metastasis at the diagnosis of cancerous meningitis, suggesting rapid development of new metastases. While pharmaco-synergy—stereotactic radiotherapy-lapatinib^[17]^—provides higher local disease control, the general condition of patients with carcinomatous meningitis determines the prognosis following whole-brain radiotherapy. In this study, the patient's prognosis with HER2 type are superior. This reason is not clear, but we think that anti-HER2 drug (such as trastuzumab and pertuzumab) molecular weight is small and can pass the blood–brain barrier. So, the anti-HER2 drug shows anti-breast cancer effects and improves the prognosis.

A study reported median survival time of 313 days with KPS ≥ 70 and 36 days with KPS ≤ 60. In this study, the median survival time of patients who discontinued whole-brain radiotherapy or received low dose was relatively short (17.5 days), suggesting poor general condition. A survival study of patients with mean survival time of 74 days identified age, time from diagnosis to palliative radiotherapy, liver metastasis, and general condition, as factors affecting the 30-day mortality.^[18]^ However, the results of the present study showed that age, time from the definitive diagnosis of breast cancer to the onset of cancerous meningitis, and the presence or absence of other metastases (e.g., liver metastasis) do not affect overall survival. In contrast, this study identified discontinuation of whole-brain radiotherapy and presence of triple-negative breast cancer as factors of poor prognosis. Therefore, caution is required in the selection of patients who receive whole-brain radiotherapy. In addition, caution is required even after whole-brain radiotherapy because the median survival time is short (i.e., 84 days) even after whole-brain radiotherapy to a dose of 30 Gy in ≥10 fractions in patients with carcinomatous meningitis from breast cancer.

This study has some limitations. First, this was a single-center study with small sample size. Second, the prognosis of two patients was unknown. High incidence of brain metastasis in patients with triple-negative breast cancer with persisting lymph node metastasis even after chemotherapy has been reported.^[19]^ However, methods to identify patients with a high risk of carcinomatous meningitis have not been established. Future research should include multicenter studies with a bigger sample size to further investigate these issues.

To conclude, this study identified discontinuation of whole-brain radiotherapy and presence of triple-negative breast cancer as poor prognostic factors.

## Author contributions

Yukinori Okada, had the original idea and collected and analyzed the data. Tatsuyuki Abe, Mio Shinozaki, Akiko Tanaka, Mariko Kpbayash checked and suggested the clinical diagnosis. Yoshihide Kanemaki guided the imaging diagnosis Yasuyuki Kojima guided the clinical diagnosis. Hiromichi Gomi, Naoki Nakamura suggested the data analysis methods and checked the data analysis results.
